# Automated Prediction of the Response to Neoadjuvant Chemoradiotherapy in Patients Affected by Rectal Cancer

**DOI:** 10.3390/cancers14092231

**Published:** 2022-04-29

**Authors:** Giuseppe Filitto, Francesca Coppola, Nico Curti, Enrico Giampieri, Daniele Dall’Olio, Alessandra Merlotti, Arrigo Cattabriga, Maria Adriana Cocozza, Makoto Taninokuchi Tomassoni, Daniel Remondini, Luisa Pierotti, Lidia Strigari, Dajana Cuicchi, Alessandra Guido, Karim Rihawi, Antonietta D’Errico, Francesca Di Fabio, Gilberto Poggioli, Alessio Giuseppe Morganti, Luigi Ricciardiello, Rita Golfieri, Gastone Castellani

**Affiliations:** 1 Department of Experimental, Diagnostic and Specialty Medicine, University of Bologna, 40138 Bologna, Italy; giuseppe.filitto@studio.unibo.it (G.F.); gastone.castellani@unibo.it (G.C.); 2 Department of Radiology, IRCCS Azienda Ospedaliera-Universitaria di Bologna, 40138 Bologna, Italy; francesca.coppola@aosp.bo.it (F.C.); arrigo.cattabriga@me.com (A.C.); adrianacocozza1992@gmail.com (M.A.C.); makoto.taninokuchi@studio.unibo.it (M.T.T.); rita.golfieri@unibo.it (R.G.); 3 SIRM Foundation, Italian Society of Medical and Interventional Radiology, 40138 Bologna, Italy; 4 eDIMES Lab, Department of Experimental, Diagnostic and Specialty Medicine, University of Bologna, 40138 Bologna, Italy; 5 INFN Bologna, 40127 Bologna, Italy; daniel.remondini@unibo.it; 6 Department of Physics and Astronomy, University of Bologna, 40127 Bologna, Italy; daniele.dallolio@unibo.it (D.D.); alessandra.merlotti2@unibo.it (A.M.); 7 Sant’Orsola-Malpighi Polyclinic, IRCCS Azienda Ospedaliero-Universitaria di Bologna, 40138 Bologna, Italy; luisa.pierotti@aosp.bo.it; 8 Department of Medical Physics, Sant’Orsola-Malpighi Polyclinic, IRCCS Azienda Ospedaliero-Universitaria di Bologn, 40138 Bologna, Italy; lidia.strigari@aosp.bo.it; 9 Medical and Surgical Department of Digestive, Hepatic and Endocrine-Metabolic Diseases, IRCCS Azienda Ospedaliera-Universitaria di Bologna, 40138 Bologna, Italy; dajana.cuicchi@aosp.bo.it (D.C.); gilberto.poggioli@unibo.it (G.P.); 10 Department of Radiation Oncology, IRCCS Azienda Ospedaliera-Universitaria di Bologna, 40138 Bologna, Italy; alessandra.guido@aosp.bo.it (A.G.); alessio.morganti2@unibo.it (A.G.M.); 11 Division of Medical Oncology, IRCCS Azienda Ospedaliera-Universitaria di Bologna, 40138 Bologna, Italy; karim.rihawi@aosp.bo.it (K.R.); francesca.difabio@aosp.bo.it (F.D.F.); 12 Pathology Unit, Department of Specialized, Experimental and Diagnostic Medicine, IRCCS Azienda Ospedaliera-Universitaria di Bologna, 40138 Bologna, Italy; antonietta.derrico@unibo.it; 13 Department of Medical and Surgical Science, University of Bologna, 40138 Bologna, Italy; luigi.ricciardiello@unibo.it

**Keywords:** radiomics, artificial intelligence, machine and deep learning, medical imaging

## Abstract

**Simple Summary:**

Colorectal cancer is the second most malignant tumor per number of deaths after lung cancer and the third per number of new cases after breast and lung cancer. The correct and rapid identification (i.e., segmentation of the cancer regions) is a fundamental task for correct patient diagnosis. In this study, we propose a novel automated pipeline for the segmentation of MRI scans of patients with LARC in order to predict the response to nCRT using radiomic features. This study involved the retrospective analysis of T_2_-weighted MRI scans of 43 patients affected by LARC. The segmentation of tumor areas was on par or better than the state-of-the-art results, but required smaller sample sizes. The analysis of radiomic features allowed us to predict the TRG score, which agreed with the state-of-the-art results.

**Abstract:**

Background: Rectal cancer is a malignant neoplasm of the large intestine resulting from the uncontrolled proliferation of the rectal tract. Predicting the pathologic response of neoadjuvant chemoradiotherapy at an MRI primary staging scan in patients affected by locally advanced rectal cancer (LARC) could lead to significant improvement in the survival and quality of life of the patients. In this study, the possibility of automatizing this estimation from a primary staging MRI scan, using a fully automated artificial intelligence-based model for the segmentation and consequent characterization of the tumor areas using radiomic features was evaluated. The TRG score was used to evaluate the clinical outcome. Methods: Forty-three patients under treatment in the IRCCS Sant’Orsola-Malpighi Polyclinic were retrospectively selected for the study; a U-Net model was trained for the automated segmentation of the tumor areas; the radiomic features were collected and used to predict the tumor regression grade (TRG) score. Results: The segmentation of tumor areas outperformed the state-of-the-art results in terms of the Dice score coefficient or was comparable to them but with the advantage of considering mucinous cases. Analysis of the radiomic features extracted from the lesion areas allowed us to predict the TRG score, with the results agreeing with the state-of-the-art results. Conclusions: The results obtained regarding TRG prediction using the proposed fully automated pipeline prove its possible usage as a viable decision support system for radiologists in clinical practice.

## 1. Introduction

According to the International Agency for Research on Cancer, colorectal cancer is the second most malignant tumor per number of deaths after lung cancer and the third per number of new cases after breast and lung cancer (up to 2020). The European Cancer Information System (ECIS) estimates that colorectal cancer accounted for 12.7% of all new cancer diagnoses and 12.4% of all cancer deaths (up to 2021).

Locally advanced rectal cancer (LARC) is considered to be a middle-stage oncologic disease, involving the entire bowel wall (stages 2, T3/T4N0) and/or the regional lymph nodes (stage 3, any T N1/N2). The current standard treatment for patients with LARC is neoadjuvant chemoradiotherapy (nCRT) followed by total mesorectal excision (TME) [1,2,3]. Currently, according to the European Society for Medical Oncology (ESMO) guidelines, magnetic resonance imaging (MRI), in combination with endorectal ultrasound, is considered the gold standard for LARC [4,5] local staging-restaging. Precise tumor and lymph node characterization using high-end imaging technology are critical for establishing rectal cancer staging to determine the need for nCRT [5].

A pathological complete response (pCR) could be achieved in 15–27% of the patients undergoing nCRT before surgery, indicating that a “wait and watch” approach might be a good or even an ideal option for this group of patients, thus avoiding the risk of surgical complications. In addition, the rate of non-responder (NR) patients, defined as patients who do not achieve tumor regression after nCRT, is reported to be between seven and 30%. Therefore, it could be useful to predict tumor response before the beginning of nCRT in order to develop a more tailored strategy such as an intensified treatment regimen, as shown in the PRODIGE23 study, which investigated the role of neoadjuvant mFOLFIRINOX before nCRT, followed by TME surgery and adjuvant computer tomography (CT) in resectable LARC [6,7,8,9,10].

The correct and rapid identification (i.e., segmentation of the cancer regions) is a fundamental task for a correct patient diagnosis. Accurate segmentation allows us to focus the analysis on the only interesting portion of the image, improving the effectiveness of feature detection. The current gold standard in medical image segmentation involves manual or semi-automated methods. However, these approaches are both time consuming and operator dependent, and are limiting factors in the reproducibility of the results [11]. Therefore, an automated and rapid approach is fundamental to overcoming these issues.

Artificial intelligence (AI) technologies such as deep learning (DL) models have been exploited with impressive results. Convolutional neural networks (CNNs) are one of the deep-learning-based approaches largely exploited for detection and segmentation purposes, especially in the field of biomedical imaging [11,12]. The state-of-the-art applications of a CNN model for rectal cancer segmentation are at an early stage of development, similar to other medical image topics [13]. Rectal cancer segmentation on MRI scans is very challenging due to the multiple issues related to the acquisition technique (low-contrast appearance of tumor regions) and to the anatomical region involved (complexity of the peri-tumor background and spread of the tumor area involved).

Different approaches have been proposed in the literature to address this task. A notable result was achieved in the study of Trebeschi et al. [11] in which the authors developed an ad hoc CNN model for fully automated localization and segmentation of rectal cancer, trained on T_2_-weighted MRI and diffusion-weighted (DWI) scans. Despite the performance of the model, the requirement of a DWI scan poses the main limitation of their application in clinical routines; DWI scans are not always acquired in clinical practice and they require ad hoc corrections due to the acquisition modality. The study of Huang et al. [12] addressed this issue; the authors proved that the same CNN architecture trained on just T_2_-weighted MRI scans could improve its segmentation performance using a tailored loss function during the model learning.

The study of Panic et al. [14] pointed out another issue related to the anatomical structure of rectal cancers (i.e., mucinous cases). Rectal cancers can roughly be split into two main classes: mucinous carcinoma and adenocarcinomas. The former appears hyperintense in T_2_-weighted MRI images while the latter appear more hypointense. The different responses to MRI acquisition pose a critical issue for the development of automated methods and excludes the use of color/contrast features for the characterization of the lesions.

Pang et al. [15] proposed a different approach to tackle the automatic segmentation task, splitting their pipeline into two phases. In the first step, the model attempted to automatically detect the location of the lesion on the T_2_-weighted MRI scans while a second model performed segmentation on the 2D regions of interest (ROIs) identified. In this case, no mucinous tumors were included in the analysis. This approach required the manual annotation of the “suspected regions” and the contouring of the lesion areas, increasing the time-cost for the clinicians.

Even with proper segmentation algorithms, the identification of relevant radiomic features is another open problem. In fact, the automated identification of the lesion areas represents only the starting point for any automated radiomic analysis or feature extraction. The importance of radiomic features in the characterization of rectal lesions has been deeply analyzed in the literature [11]. The possibility of quantitatively analyzing the phenotypical characteristics of the tumors provides a significant benefit for patient diagnosis and prognosis.

In the studies of Bulens et al. [16] and Pang et al. [4], the authors proved the effectiveness of radiomic features in predicting a pathologic complete response (pCR) in patients affected by locally advanced rectal cancer after neoadjuvant chemoradiotherapy. Additional correlations between radiomic features and interesting clinical scores were identified in the studies of Soomro et al. [17] and Li et al. [18]. They proved the possibility of monitoring tumor evolution using quantitative measurements extracted from T_2_-weighted MRI scans, predicting the stage malignancy score and the tumor regression grade (TRG), respectively.

In the majority of cases, radiomic features have been extracted by experts, starting from a (semi-)manual segmentation of the lesion areas. The possibility of carrying out a completely automated analysis of rectal samples, starting from raw T_2_-weighted MRI scans to predict clinical outcomes, still remains limited.

In this study, the authors proposed a novel automated pipeline for the segmentation of MRI scans of patients with LARC in order to predict the response to nCRT using radiomic features. 

The TRG score was used to evaluate the clinical outcome. The classic TRG score ranges from 0 to 5, using discrete integer values. A surgical resection histopathological report from the samples was obtained for all the patients at the time of TME. Consequently, following the TRG staging system, the patients were initially split into four groups, according to the American Joint Committee on Cancer (AJCC) [19]:

- TRG0: no viable cancer cells (pathological complete response);
- TRG1: single or small groups of tumor cells (moderate response);
- TRG2: residual cancer outgrown by fibrosis (minimal response); and
- TRG3: minimal or no tumor cells killed (poor response).

The patients were finally clustered into two groups, based on the TRG stage: Group 1 (i.e., patients with TRG ∈ [0, 1], who obtained a moderate response or a pCR), and Group 2 (i.e., patients with TRG ∈ [2, 3], who obtained a poor or minimal response).

The study was developed and validated on MRI scans provided by the IRCCS Sant’Orsola-Malpighi Polyclinic of the University of Bologna.

## 2. Materials and Methods

### 2.1. Patient Selection

This study involved the retrospective analysis of T_2_-weighted MRI scans of 43 patients affected by LARC. Only patients who received a diagnosis of LARC in the authors’ institution and underwent primary MR staging were selected by the Radiology Unit of the IRCCS Sant’Orsola-Malpighi Polyclinic from January 2018 to the end of December 2020. The study was conducted according to the guidelines of the Declaration of Helsinki and was approved by the Institutional Review Board (or Ethics Committee) of IRCCS University Hospital di Bologna (protocol code no. 842/2020/Oss/AOUBo). The subjects underwent a neoadjuvant chemoradiotherapy regimen that involved long-course by intensity modulated RT (IMRT) delivered using an Elekta Sinergy Linac with photon energies of 6–15 MV with a total dose of 50.4 Gy (1.8 Gy/fraction) and concurrent oral capecitabine (825 mg/m^2^ BID).

This study considered all 43 patients for segmentation and the 39 who were treated with long-course nCRT followed by TME for radiomic feature extraction, according to the exclusion criteria. The radiomic analysis excluded patients who did not undergo standard nCRT according to the directions of the authors’ multidisciplinary team, TME patients whose pathological report did not include TRG grading, patients whose MR study was incomplete and did not provide sufficient information for proper tumor staging, and/or those who were affected with imaging artifacts, which would have impaired the radiomic analysis (refer to Table 1).

A total of 60% of the selected patients were male, and the age distribution was 48/70/89 (min/mean/max). Rectal cancer volumes were labelled by an expert radiologist (with more than 10 years of experience). The manual annotations consisted of sets of 2D coordinates related to the tumor area boundaries in each patient slice. For each patient, experts indicated the TRG score as degree of response after nCRT.

According to the European Society of Gastrointestinal and Abdominal Radiology (ESGAR) guidelines for rectal cancer primary staging, a MRI pelvic scan with a 1.5 T MRI (GE Healthcare) was performed for all patients [20]. The protocol included three T_2_-weighted fast spin echo (FSE) sequences: a sagittal high-resolution sequence, an oblique-axial high-resolution sequence orthogonal to the tumor axis, and an oblique coronal high-resolution sequence parallel to the tumor axis. Diffusion-weighted images were obtained in the axial planes using echo-planar imaging (EPI) sequences at three b-values (b0, b600, and b1000 s/mm^2^), and restriction of the diffusion was quantified using the apparent diffusion coefficient (ADC) value. In addition, a T_1_ axial sequence of the entire pelvis was acquired. Bowel cleansing was performed with two days of a low-fiber diet and the oral administration of Macrogol-Na-K 14 gr the day before the study.

### 2.2. Pipeline Overview

**Figure 1:**
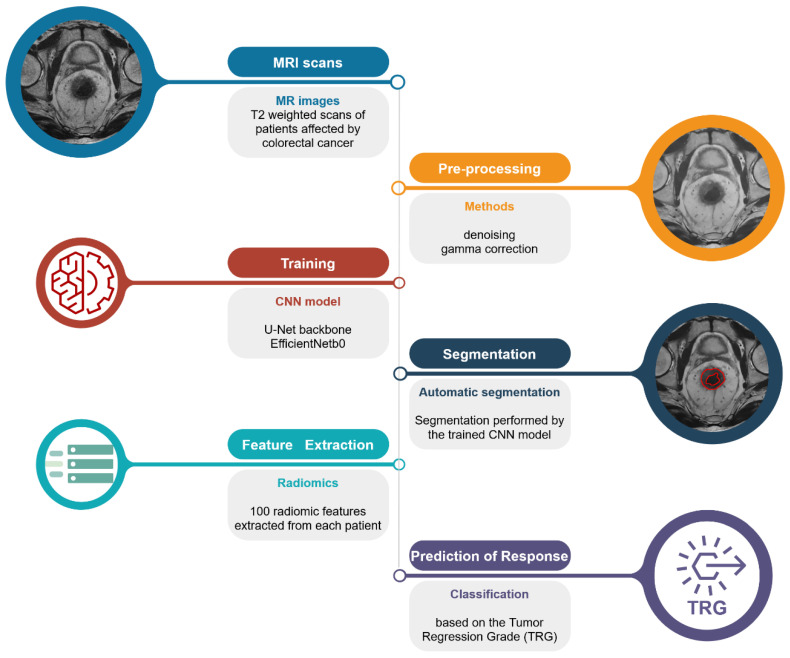
Schematic representation of the proposed pipeline. From the top left: raw T_2_-weighted MRI scan; pre-processed image using denoising algorithm and gamma correction for the remotion of possible confounders; segmentation of the lesion areas using the authors’ CNN model; extraction of the radiomic features from the areas identified by the CNN model; and prediction of the TRG score.

The automated pipeline proposed in this study (Figure 1) can be divided into three steps: (1) segmentation of the lesion areas; (2) estimation of the radiomic features; and (3) prediction of the TRG score.

### 2.3. Rectal Segmentation

Tumor area segmentation is a crucial preprocessing step for the following feature extraction task, allowing the algorithms to be the focus of only the area of interest. Several models based on complex deep learning models have already been proposed in the literature to address this task; however, the top-performing models do not provide the internal model parameters, requiring a full re-training of the model architecture. Therefore, a tailored training procedure was performed.

The entire set of MRI slices (resolution 512 × 512) was scaled in the range [0, 1]. An initial non-local mean filter was applied on the MRI scans to reduce possible noise sources, and a gamma correction was applied to improve the image contrast. The use of a non-local filter for image smoothing allowed us to preserve the details of the original image, removing the noise components. The gamma correction algorithm allowed us to enhance the contrast of the image, improving the brightness of the darker areas; in the pipeline in the present study, a gamma equal to 0.6 was used. Both algorithms were applied slice-by-slice for each patient’s images.

The entire dataset included more than 1070 MRI slices; however, only 480 of them involved lesion areas. Training and testing of the segmentation model were carried out considering only the slices with at least one pixel belonging to the tumor region. The manual annotations, provided as sets of (x, y) coordinates of the lesion boundary, were converted into binary images, labelling the pixels inside the tumor as 1, and the background as 0.

For the lesion area segmentation, a 2D U-Net-like neural network model was trained. EfficientNetb021 architecture [21] was used as a backbone for the classic 2D U-Net to improve the training efficiency of the model.

The entire dataset of 43 patients was organized into two train/test disjoint subsets (30/13 patients, respectively). A data augmentation procedure including random vertical and/or horizontal flip was applied to the training set to improve the robustness of the model.

The segmentation performances were evaluated by using class balancing. In the current task, it can be reasonably assumed that the number of pixels included in the lesion area would be very few compared to the background pixels. In this case, the standard metric functions needed to consider an unbalanced number of samples. Different loss functions suitable for highly imbalanced data were tested. A possible solution for overcoming this issue is given by the Dice score coefficient (*DSC*, or Sørensen–Dice coefficient), which measures the similarity of two samples (i.e., the output mask and the ground truth) as:

$$DSC=\frac{2\times TP}{2\times TP+FN+FP}$$

where *TP*, *FP*, and *FN* are the True Positive, False Positive, and False Negative scores, respectively. The same score has already been used in other studies [11,12,14,15], thus guaranteeing a direct comparison of the present results with the state-of-the-art results.

The segmentation was performed slice-by-slice for each patient using the trained U-Net model, obtaining the related lesion areas as binary masks. The parameters used for the training procedure are reported in the Supplementary Materials.

The model parameters obtained regarding the reproducibility of the results are publicly available on Github [22].

All of the simulations were carried out using a 64-bit workstation machine (8 GB RAM memory and one CPU Apple M1, with eight cores, and a GPU with eight graphics core).

### 2.4. Feature Extraction

The proposed pipeline was applied to the 43 patients of the IRCCS Sant’Orsola-Malpighi Polyclinic of Bologna. The radiomic feature extraction was performed on the automatically identified lesion areas. The extracted features included morphological and texture-based scores. Radiomic features considered both 2D and 3D medical samples; they can be subdivided into 1st order statistics (18 features), 2D/3D shape-based features (14 features), gray level co-occurrence matrix statistics (22 features), gray level size zone matrix statistics (16 features), gray level run length matrix statistics (16 features), and gray level dependence matrix statistics (14 features). For each patient, a total of 100 radiomic features were extracted.

The entire set of features was extracted using the Pyradiomics library [23].

### 2.5. TRG Prediction

The entire set of features was standardized to a common range and rescaled using their mean values, normalizing them according to their variance.

Using a principal component analysis (PCA), the number of features was reduced by removing co-linearities and non-informative components. In this way, the problem of dimensionality was reduced by excluding useless components from the analysis, based on the percentage of explained variance.

The processed set of features was then used in a support vector classifier (SVC) model; the parameters used for the analysis are reported in the Supplementary Materials.

The available patients were organized into two disjointed subsets, namely training and test sets, using a 10-fold cross validation. The model was trained on a subset (90%) of samples and its predictions were compared to the ground truth provided by the corresponding test set (10%).

Agreement between the predicted response and the correct response was measured using several metrics, in other words:

$$Precision=\frac{TP}{TP+FP}$$

$$Recall=\frac{TP}{TP+FN}$$

$$F_{1}\;score=\frac{2TP}{2TP+FP+FN}$$

$$MCC=\frac{TP\times TN-FP\times FN}{\sqrt{\left(TP+FP\right)\left(TP+FN\right)\left(TN+FP\right)\left(TN+FN\right)}}$$

in which *TP*, *TN*, *FP*, and *FN* are the True Positive, True Negative, False Positive, and False Negative scores, respectively. The authors used the results obtained by Li et al. [18] as a benchmark for the efficiency of their pipeline. The area under the curve (AUC) score was added as an extra-metric for the comparison.

The TRG score was not included in the entire set of patients and, therefore, only the 39 patients with an estimated TRG score were selected for the analysis. Due to the small set of patients available, the authors binarized the TRG into two classes using TRG = 2 as a hard threshold. Patients with a complete-like response to nCRT (TRG ∈ [0, 1]) were included in the first class (class 0, 16/39 patients, 41%); patients with a moderate-like response (TRG ∈ [2, 3]) were included in the second class (class 1, 23/39 patients, 59%). Cases with a TRG > 3 were not included in this study.

The scikit-learn [24] python package was used for the implementation of all the analyses.

## 3. Results

### 3.1. Rectal Segmentation

**Figure 2:**
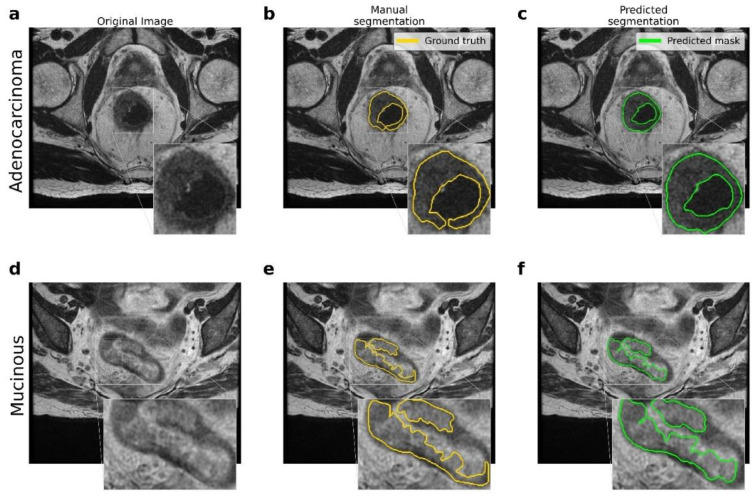
Comparison between the ground truth and the results obtained by the proposed pipeline for the lesion segmentation. (**a**–**d**) Original MRI scans for *adenocarcinoma* and *mucinous* cases. (**b**–**e**) Ground truth obtained by manual segmentation performed by experts. (**c**–**f**) Predicted segmentation obtained by the proposed U-Net model.

Figure 2 shows the resulting segmentations obtained by the U-Net model. The comparison between the original (raw) image, the manual segmentation performed by an expert radiologist, and the segmentation resulting from the application of the proposed U-Net model are reported. In addition, there were significant differences between the two types of lesions involved, namely *adenocarcinoma* and *mucinous* carcinoma: in T_2_-weighted images, mucinous rectal cancers appeared as significantly hyperintense compared to adenocarcinoma neoplasms.

Table 2 reports the comparison between the proposed pipeline and other state-of-the-art models, expressed in terms of the *DSC* score. The difference in terms of number of patients involved in the different studies and the inclusion/exclusion of mucinous cases should be stressed; both of these factors can affect the performance of the CNN models.

### 3.2. TRG Prediction

The radiomic feature extraction procedure was applied to 39/43 samples for which the TRG score was noted. The radiomic features considering the ROIs obtained by the present segmentation model were extracted.

**Figure 3:**
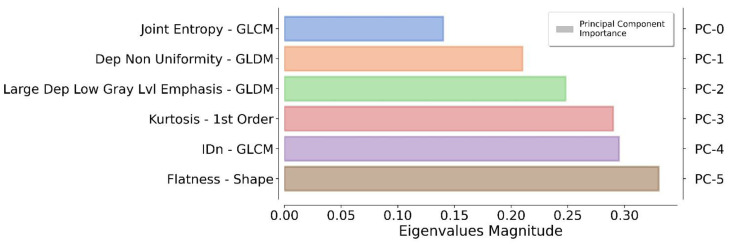
Ranking of the top informative features identified by the proposed pipeline. For each principal component (on the **right**), the original feature (on the **left**) that contributes the most to the related principal component is reported.

The application of a PCA highlighted the fact that only six components were representative of the 90% of the total variance. The authors reflected on the importance of each feature by monitoring the magnitude of the corresponding eigenvector values (higher magnitude leads to greater importance). In this way, redundant features can be filtered out by ranking them according to their informative power (Figure 3).

This core set of features was used to train the SVC model to predict the TRG score. Five hundred 10-fold cross validations were carried out to obtain a distribution of the performance metrics. The resulting scores, expressed in terms of mean ± standard deviation, are reported in Table 3.

The results obtained from the authors’ pipeline were compared with the state-of-the-art results proposed by Li et al. [18]. The comparison of the results is reported in Table 4 including the information related to the two pipelines.

## 4. Discussion

In this study, an automated pipeline for the identification, segmentation, and analysis of rectal cancer MRI scans was introduced. The CNN model proposed in this study obtained a performance compatible or superior to a set of equivalent state-of-the-art deep learning models. The proposed U-Net-like model obtained a *DSC* = 0.74, outperforming the state-of-the-art results and including one mucinous case in the prediction.

The study of Panic et al. [14] pointed out how mucinous cases can considerably affect the segmentation performance of a CNN model. In the present study, the same issue was dealt with; however, the proposed pipeline seemed to partially overcome this problem with a robust segmentation model. The limited number of samples involved in this study does not allow for generalizing of this conclusion, as a result, additional analyses are required.

A second source of issues was pointed out by the study of Trebeschi et al. [11] regarding the quality of manual annotations; in this study, samples manually annotated and checked by an expert radiologist to improve the reliability of the training data were used. Furthermore, the network architecture and losses could be discriminant factors for rectal segmentation performance due to the complexity of the rectal region on MRI scans, as discussed by Huang et al. [12]. In this study, the *DSC* was used as a loss function to control the unbalancing of the pixel classes related to the anatomical regions involved.

The proposed pipeline seemed to circumvent all these issues and demonstrated that it was suitable for rectal cancer segmentation. This was apparent from the improvement in the *DSC*, the inclusion of one mucinous case, the use of single MRI (T_2_-weighted) acquisitions, and the use of relatively simple neural network architecture (Table 1). These results were obtained despite the smaller dataset size compared to other studies.

The main limit of this work was certainly given by the small dataset used, which could not guarantee a robust generalization of the proposed model. However, the cross validation used for the training and test of the model performances aimed to estimate the generalization capability of the model. The variability of the *DSC* score obtained on the test set (Table 2) confirmed the robustness of our model. The main drawback of this approach is given by the single-center nature of the study, which limits the generalizability to other centers.

The radiomic pipeline developed for the prediction of the TRG obtained significant results in terms of precision, recall, and F1-score, equal to 0.70, 0.79, and 0.74 for TRG ∈ [0, 1], and 0.84, 0.77 and 0.80 for TRG ∈ [2, 3], respectively. It should be noted that the results were obtained using a 10-fold cross validation of the data (i.e., predicting the TRG score on a blind set of data) as an average score using 500 train/test subdivisions. The robustness of the results was confirmed by an average Matthew’s correlation coefficient (*MCC*) score equal to 0.55.

The results for the prediction of the TRG score, obtained by the authors’ pipeline, were compatible with the state-of-the-art results (Table 4), despite the low number of samples involved in this study for the model training. It should be noted that the proposed pipeline started from an automated identification of the lesion areas while the Li et al. [18] approach, a benchmark for the present model, used manual annotation. The extraction of features from a pixel-perfect tumor area guarantees the robustness of the radiomic measurements, avoiding the possible confounders introduced by automated models. The automated segmentation obtained in this study allowed for the extraction of a sufficiently robust set of radiomic features to overcome this issue.

Future studies might also consider radiomic features extracted by post-nCRT restaging MRI and compare them both with primary-staging MRI and with the TRG score itself.

## 5. Conclusions

The results obtained by the proposed fully automated pipeline proved its possible usage in clinical practice after additional validations for both image segmentation and for the prediction of response as a viable support system for radiologists. Although encouraging, the results in the present study must be validated by a multi-center study with a larger cohort of patients.

## Figures and Tables

**Table 1:** Baseline characteristics of the patients included in the analysis after receiving the same neoadjuvant chemoradiotherapy regimen. Abbreviations: ECOG-PS: “Eastern Cooperative Oncology Group performance status”; TRG: tumor regression grade; cT and cN: “clinical tumor stage” and “clinical node stage”, respectively, according to the TNM staging of rectal cancer.

Characteristics	Responder TRG 0–1 (*n* = 16)	Non-Responder TRG 2–3 (*n* = 23)	Total (*n* = 39)
Sex, males/females, *n*	12/4	15/8	27/12
%	75/25	65/35	69/31
Median age (range), years	66 (33–85)	63 (46–82)	65 (33–85)
ECOG-PS 0	13 (81%)	19 (82%)	32 (82%)
ECOG-PS 1	2 (12.5%)	3 (13%)	5 (13%)
ECOG-PS 2	1 (6.5%)	1 (5%)	2 (5%)
cT	T2 2 (12.5%)	T2 1 (4%)	T2 3 (8%)
	T3 13 (81%)	T3 17 (74%)	T3 30 (77%)
	T4 1 (6.5%)	T4 5 (22%)	T4 6 (15%)
cN	N− 3 (19%)	N− 5 (20.0%)	N− 8 (20%)
	N+ 13 (81%)	N+ 18 (80:0%)	N+ 31 (80%)

**Table 2:** Comparison between state-of-the-art pipelines for the automated segmentation of rectal cancer. All the results of the comparison are expressed in terms of the *DSC* score. The last column reports the results obtained by the proposed pipeline regarding the data involved in the present study. In relation to the study of Trebeschi et al., the *DSC* score obtained by the two experts involved in the study (i.e., expert 1 (*) and expert 2 (**) is reported).

Trebeschi et al. [11]	Panic et al. [14]	Yi-Jie Huang et al. [12]	Xiaoling Pang et al. [15]	The Authors’ Pipeline
* *DSC* = 0.68 ** *DSC* = 0.70	*DSC* = 0.58	*DSC* = [0.66–0.72]	*DSC* = 0.66	*DSC* = [0.73–0.75]
140 patients	33 patients (5 mucinous)	64 patients (adenocarcinoma)	275 patients (no mucinous)	43 patients (1 mucinous)
MRI T_2_w + DWI	MRI T_2_w + DWI	MRI T_2_w	MRI T_2_w	MRI T_2_w
Custom CNN	Custom CNN	Custom ensemble of CNN and losses	U-Net	U-Net + EfficientNetb0 backbone

**Table 3:** The results obtained by the proposed pipeline regarding 500 different 10-fold cross validations for predicting the TRG score. For each metric, the average value obtained ± standard deviation is reported. The number of samples for each class is reported in the first column. The global Matthews correlation coefficient (*MCC*) is reported in the last column.

	Support	*Precision*	*Recall*	*F*_1_-*Score*	*MCC*
TRG ∈ [0, 1]	13 (41%)	0.70 ± 0.05	0.79 ± 0.07	0.74 ± 0.05	0.55 ± 0.09
TRG ∈ [2, 3]	19 (59%)	0.84 ± 0.05	0.77 ± 0.05	0.80 ± 0.04	

**Table 4:** Comparison between the authors’ pipeline and the state-of-the-art pipeline proposed by Zhihui Li et al. for the automated prediction of the TRG score. For both pipelines, the number of patients involved in the study, the segmentation procedure (manual/automated) used for the lesion ROI identification, the radiomic pipeline developed, and the resulting prediction scores expressed in terms of AUC are specified.

	Zhihui Li et al. (2021) [18]	The Authors’ Pipeline
Number of patients	80 patients	39 patients
Segmentation	Manual	Automated
Radiomic pipeline	Leave-One-Out cross validation LASSO (3 components) Classifier: ○ Logistic Regression (LR) ○ Random Forest (RF) ○ Decision Tree (DT) ○ K-Nearest Neighbor (KNN)	Leave-One-Out cross validation PCA (6 components) Classifier: ○ Support Vector Classifier (SVC)
Area Under the Curve	AUC = [0.76, 0.93, 0.63, 0.84] *	AUC = 0.89

* According to the different classifiers.

## Data Availability

The study was based on a mix of public data and data collected in loco. The data collected in loco are available on request due to restrictions on privacy (European GDPR). The pre-trained model and parameters used for the image segmentation are available in the repository, *img-segm* (https://github.com/giuseppefilitto/img-segm) (Accessed on 28 February 2022).

## Supplementary Materials

The following supporting information can be downloaded at: https://www.mdpi.com/2072-6694/14/9/2231/s1, File S1: The U-Net model discussed in this study was implemented using the Tensorflow [25] python package.
