# Supplementary material for: Automated Prediction of the Response to Neoadjuvant Chemoradiotherapy in Patients Affected by Rectal Cancer
Source: Cancers (Basel). 2022 Apr 29;14(9):2231. doi: 10.3390/cancers14092231 (PMC9100060; doi:10.3390/cancers14092231)
Supplement: Supplementary file 1 [file cancers-14-02231-s001.zip › cancers-1641386-supplementary.pdf]

Automated Prediction of the Response to Neoadjuvant Chemoradiotherapy in Patients Affected by Rectal Cancer

Giuseppe Filitto [1,†], Francesca Coppola [2,3,†], Nico Curti [4,5,*], Enrico Giampieri [4,*], Daniele Dall'Olio [6], Alessandra Merlotti [6], Arrigo Cattabriga [2], Maria Adriana Cocozza [2], Makoto Taninokuchi Tomassoni [2], Daniel Remondini [5,6], Luisa Pierotti [7], Lidia Strigari [8], Dajana Cuicchi [9], Alessandra Guido [10], Karim Rihawi [11], Antonietta D'Errico [12], Francesca Di Fabio [11], Gilberto Poggioli [9], Alessio Giuseppe Morganti [10], Luigi Ricciardiello [13], Rita Golfieri [2,‡] and Gastone Castellani [1,‡]

File S1:

The U-Net model discussed in this study was implemented using the Tensorflow [25] python package. To improve learning efficiency, the MRI scans were scaled into the range [0, 1] before being fed into the models. The training procedure was carried out using batches of size 4 scans, for 150 epochs. The model was trained using the Adam optimization algorithm with a learning rate of 10^{-3}. The model was trained using 391 MRI slices (training set) and validated on 97 images (validation set). The SVC classifier used to predict the TRG was implemented using the scikit-learn [24] python package. The model was trained using a regularization parameter equal to 100 and a radial basis function kernel. The code developed for the reproducibility of the results is publicly available on Github [22].
